# Supplementary figures and images for: Detection of significant antiviral drug effects on COVID-19 with reasonable sample sizes in randomized controlled trials: A modeling study
Source: PLoS Med. 2021 Jul 6;18(7):e1003660. doi: 10.1371/journal.pmed.1003660 (PMC8259968; doi:10.1371/journal.pmed.1003660)

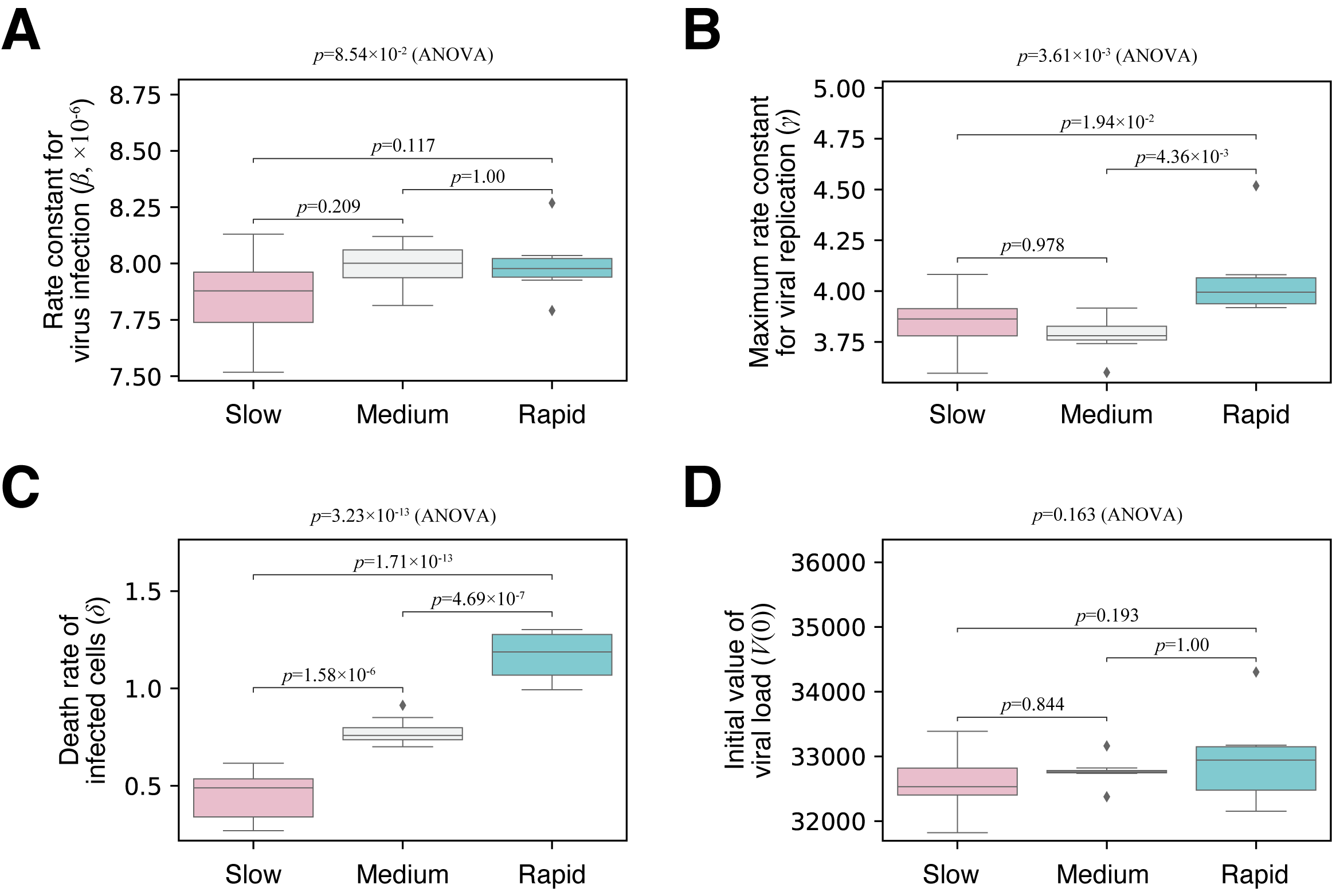

Supplement: S1 Fig — Boxplots of estimates of (A) the rate constant for virus infection, β; (B) the maximum rate constant for viral replication, γ; (C) the death rate of virus-producing cells, δ; and (D) viral load at symptom onset, V(0), respectively. Estimated parameter distributions between the 3 groups with different viral load dynamics (slow, medium, and rapid viral load decay groups) were compared by ANOVA. Pairwise comparison was subsequently performed using Student t test. The p-values of the pairwise Student t test were adjusted by the Bonferroni correction. The underlying data for this figure can be found in S2 Table. (TIF) [file pmed.1003660.s001.tif]

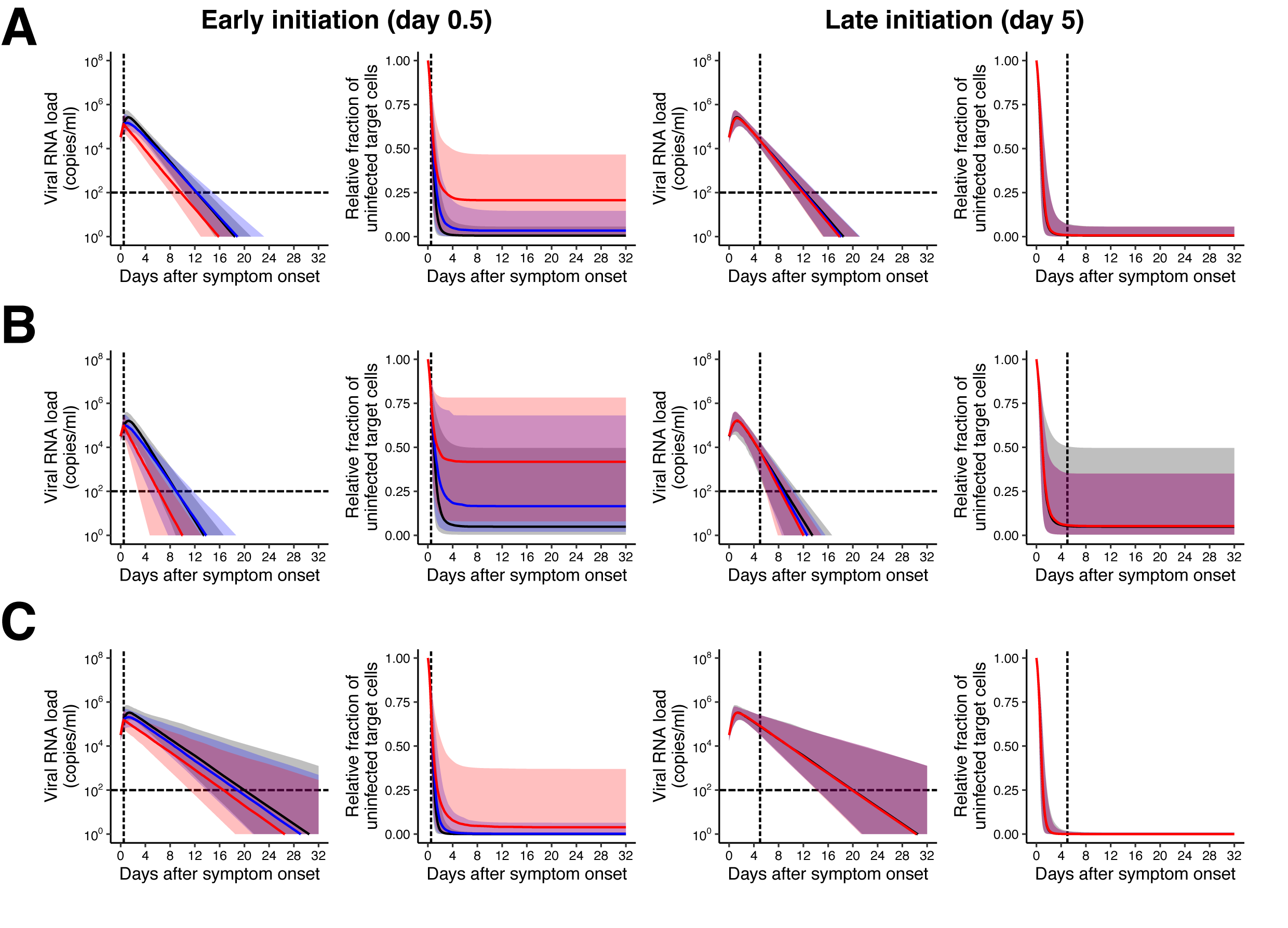

Supplement: S2 Fig — The antiviral treatment was assumed to be initiated after 0.5 or 5 days (named early and late initiations, respectively) from symptom onset with 99% and 50% inhibition rates (named high and low antiviral effects, respectively) for patients with (A) medium, (B) rapid, and (C) slow viral load decay. Left and right panels in each group show the viral loads, V(t), and the relative fraction of uninfected target cells, f(t). The black and colored solid lines correspond to the mean of the values without and with the therapies (red: high, blue: low antiviral effects), respectively. The shadowed regions correspond to the 95% predictive intervals. The underlying data for this figure can be found in S5 Data. (TIF) [file pmed.1003660.s002.tif]

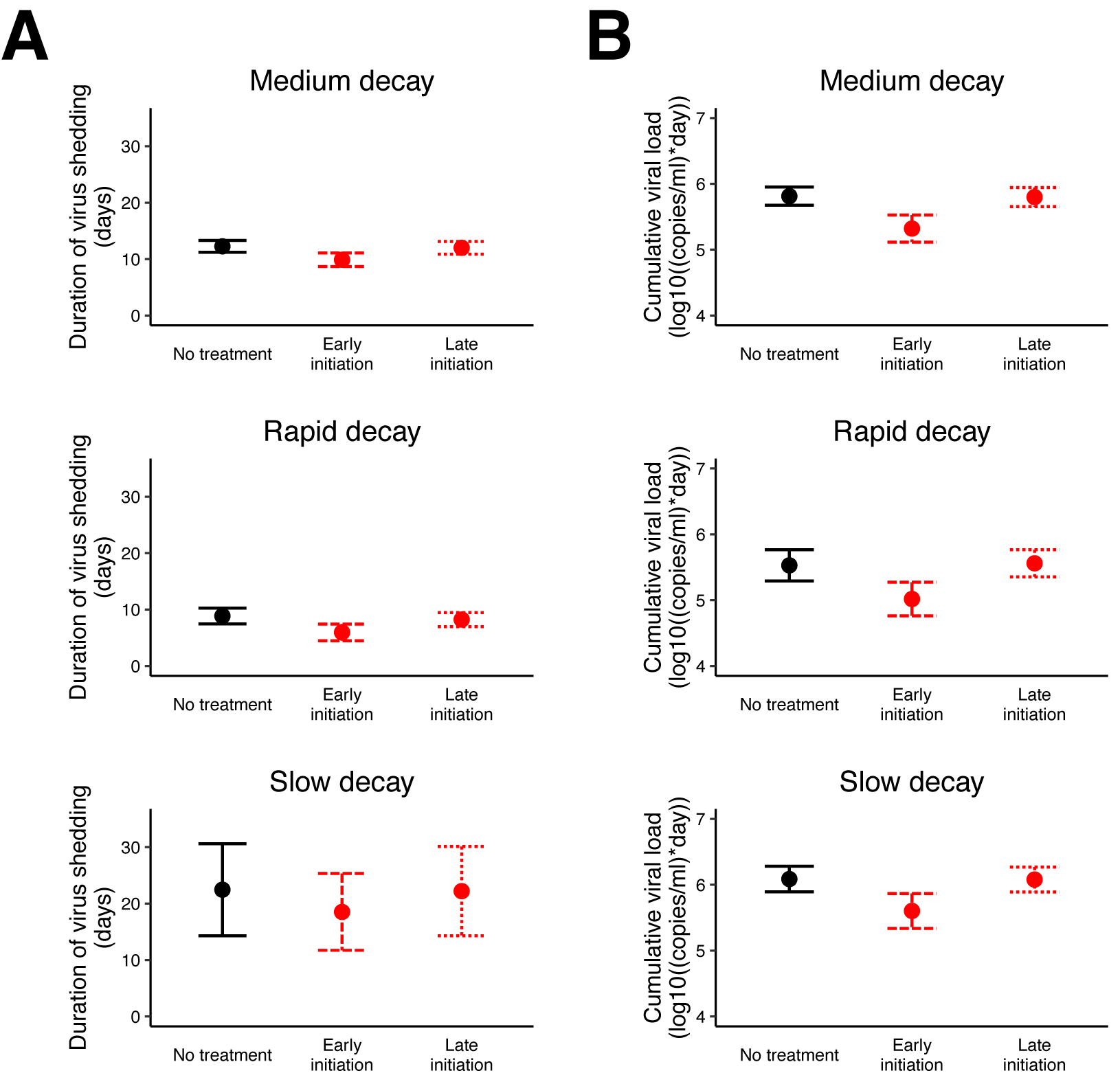

Supplement: S3 Fig — (A) Duration of virus shedding and (B) cumulative viral load of SARS-CoV-2 for different types of patients (medium, rapid, and slow decay) with and without treatment. “Early initiation” and “Late initiation” mean the early and the late treatment initiation (0.5 or 5 days after symptom onset). The dots and error bars represent the mean and the standard deviation. The underlying data for this figure can be found in S3 Data. SARS-CoV-2, Severe Acute Respiratory Syndrome Coronavirus 2. (TIF) [file pmed.1003660.s003.tif]

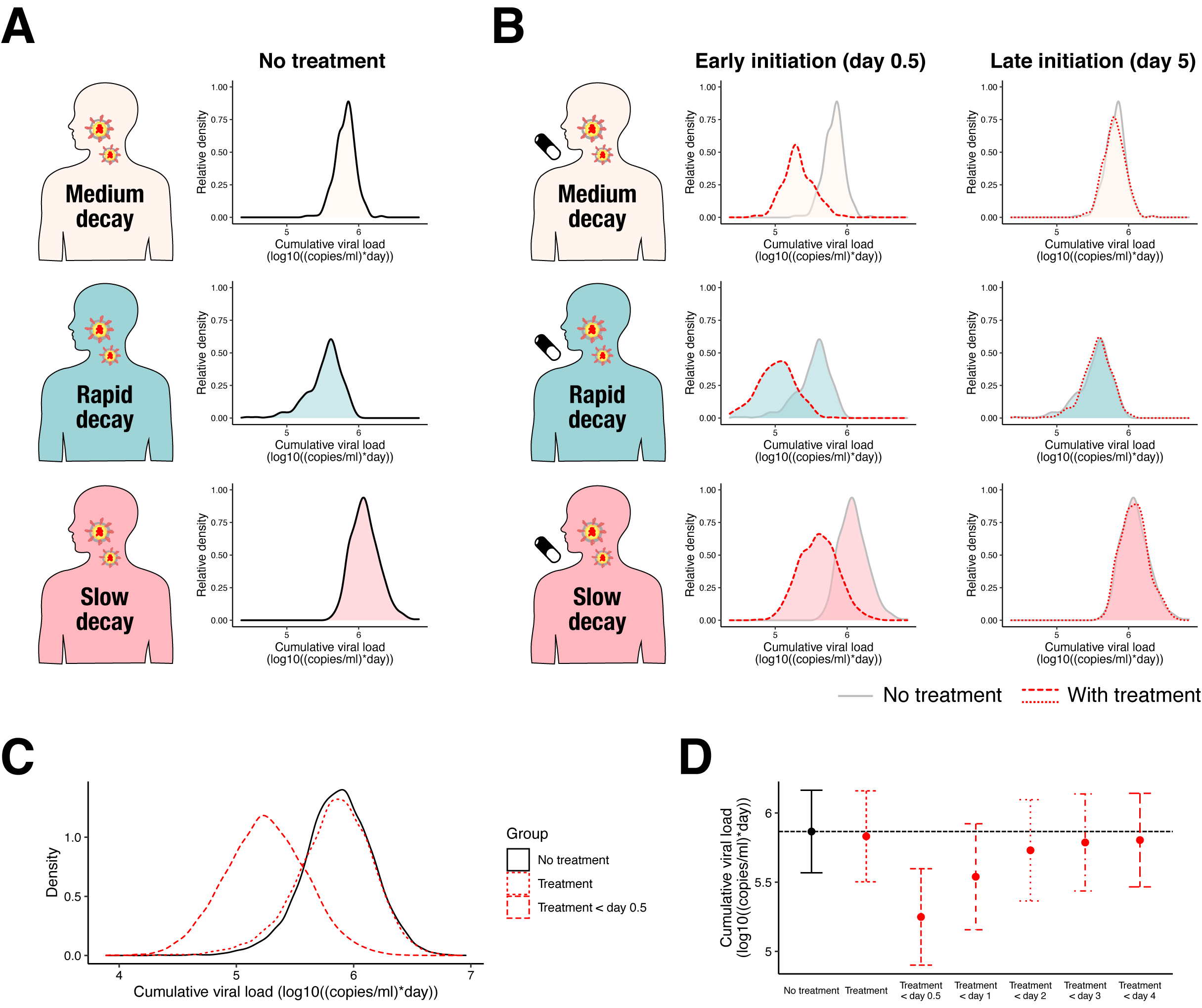

Supplement: S4 Fig — (A) The relative density distributions of cumulative viral load for the 3 groups (light pink: medium decay group, light blue: rapid decay group, and pink: slow decay group) without treatment obtained through simulation. (B) The relative density distributions of cumulative viral load (in log scale) for the 3 groups under antiviral treatment with different inhibition rate and different timing of treatment initiation. The left 3 panels are when antiviral treatment is initiated at 0.5 days (“Early initiation”) since symptom onset. The red dotted line corresponds to the distribution with 99% inhibition rate. The distribution without treatment is shown in the back for comparison. The right 3 panels are when antiviral treatment is initiated at 5 days (“Late initiation”) since symptom onset. The distributions are represented as “relative density” to reflect different proportions of the 3 groups. (C) The distributions of cumulative viral load for patients without and with treatment are shown in black and red curves, respectively. The red dotted curve is when all patients are included regardless of the timing of recruiting and treatment initiation. The red dashed curve is the case for patients who were recruited and who received treatment within 0.5 days since symptom onset only. (D) Means and standard deviation of the cumulative viral load under different inclusion criteria (not treated, included regardless of the timing of recruiting and treatment initiation, and early treatment initiation [within 0.5, 1, 2, 3, and 4 days since symptom onset]) are shown. The underlying data for this figure can be found in S3 and S4 Data. (TIF) [file pmed.1003660.s004.tif]
