# Supplementary material for: Detection of significant antiviral drug effects on COVID-19 with reasonable sample sizes in randomized controlled trials: A modeling study
Source: PLoS Med. 2021 Jul 6;18(7):e1003660. doi: 10.1371/journal.pmed.1003660 (PMC8259968; doi:10.1371/journal.pmed.1003660)
Supplement: S1 Table — Current major clinical studies for antiviral treatment of SARS-CoV-2 (as of May 22, 2020) were investigated and their information were summarized. SARS-CoV-2, Severe Acute Respiratory Syndrome Coronavirus 2. (DOCX) [file pmed.1003660.s005.docx]

| Treatment | Study design^$^ | Timing of initiation since onset (days) | Sample size (control/treatment) | Primary outcome^*^ | Effective^#^ | Reference | Peer-reviewed |
| --- | --- | --- | --- | --- | --- | --- | --- |
| Lopinavir/ritonavir | RCT | 13 (IQR: 11-16) | 99/100 | 1 | No | (*1*) | Yes |
| Remdesivir | RCT | 10 (IQR: 9-12) | 79/158 | 1 | No | (*2*) | Yes |
| Remdesivir | RCT | 9 (IQR: 6-12) | 521/538 | 1 | Yes | (*3*) | Yes |
| Hydroxychloroquine | RCT | Not reported | 31/31 | 1 | Yes | (*4*) | No |
| Hydroxychloroquine | RCT | 16.6 (SD: 10.5) | 75/75 | 2 | No | (*5*) | Yes |
| Lopinavir/ritonavir | RCT | 3.5 (IQR: 2-6) | 17/34 | 2 | No | (*6*) | No |
| Arbidol | RCT | 6 (IQR: 2-8) | 17/35 | 2 | No | (*6*) | No |
| Remdesivir | OS | 12 (IQR: 9-15) | 53 | 1,3 | - | (*7*) | Yes |
| Hydroxychloroquine | OS | 4.1 (SD: 2.6) | 16/20 | 2 | Yes | (*8*) | Yes |
| Hydroxychloroquine | OS | Not reported | 565/811 | 1,3 | No | (*9*) | Yes |
| Hydroxychloroquine and azithromycin | OS | 4.9 (SD: 3.6) | 80 | 1,2 | - | (*10*) | Yes |
| Meplazumab | OS | Not reported | 11/17 | 2 | Yes | (*11*) | No |

$ RCT: randomized control trial, OS: observational study

* 1. Clinical improvement/recovery, 2. Duration of virus shedding, 3. Mortality.

^#^ Whether the primary outcome is statistically significantly different between control and treatment group. ‘-’ denotes no statistical test was performed because it is a single-arm study (i.e., no control group).

Reference

1. B. Cao *et al.*, A Trial of Lopinavir-Ritonavir in Adults Hospitalized with Severe Covid-19. *N Engl J Med*, (2020).

2. Y. Wang *et al.*, Remdesivir in adults with severe COVID-19: a randomised, double-blind, placebo-controlled, multicentre trial. *The Lancet*, (2020).

3. J. H. Beigel *et al.*, Remdesivir for the Treatment of Covid-19 — Preliminary Report. *New England Journal of Medicine*, (2020).

4. Z. Chen *et al.*, Efficacy of hydroxychloroquine in patients with COVID-19: results of a randomized clinical trial. *medRxiv*, 2020.2003.2022.20040758 (2020).

5. W. Tang *et al.*, Hydroxychloroquine in patients with mainly mild to moderate coronavirus disease 2019: open label, randomised controlled trial. *BMJ* **369**, m1849 (2020).

6. Y. Li *et al.*, Efficacy and Safety of Lopinavir/Ritonavir or Arbidol in Adult Patients with Mild/Moderate COVID-19: An Exploratory Randomized Controlled Trial. *Med (N Y)* **1**, 105-113.e104 (2020).

7. J. Grein *et al.*, Compassionate Use of Remdesivir for Patients with Severe Covid-19. *N Engl J Med*, (2020).

8. P. Gautret *et al.*, Hydroxychloroquine and azithromycin as a treatment of COVID-19: results of an open-label non-randomized clinical trial. *Int J Antimicrob Agents*, 105949 (2020).

9. J. Geleris *et al.*, Observational Study of Hydroxychloroquine in Hospitalized Patients with Covid-19. *N Engl J Med* **382**, 2411-2418 (2020).

10. P. Gautret *et al.*, Clinical and microbiological effect of a combination of hydroxychloroquine and azithromycin in 80 COVID-19 patients with at least a six-day follow up: A pilot observational study. *Travel Med Infect Dis* **34**, 101663 (2020).

11. H. Bian *et al.*, Meplazumab treats COVID-19 pneumonia: an open-labelled, concurrent controlled add-on clinical trial. *medRxiv*, 2020.2003.2021.20040691 (2020).
