# Supplementary material for: Detection of significant antiviral drug effects on COVID-19 with reasonable sample sizes in randomized controlled trials: A modeling study
Source: PLoS Med. 2021 Jul 6;18(7):e1003660. doi: 10.1371/journal.pmed.1003660 (PMC8259968; doi:10.1371/journal.pmed.1003660)
Supplement: S2 Table — The conditional modes of the individual parameters for each patient were estimated as empirical Bayes estimates and summarized. The patient type was based on the 3 groups identified by the hierarchical clustering of the reconstructed daily viral load data. (DOCX) [file pmed.1003660.s006.docx]

| Patient ID | $\gamma$ (day^-1^) | $\beta$ ((RNA copies/ml)^-1^day^-1^) | $\delta$ (day^-1^) | $V\left( 0 \right)$ (RNA copies/ml) | Patient type |
| --- | --- | --- | --- | --- | --- |
| Germany | | | | |  |
| 1 | $3.74$ ($2.25-5.99$) | $8.12$($5.48-11.2$)$\times{10}^{-6}$ | $0.75$ ($0.68-0.93$) | $3.32$($1.84-5.70$)$\times{10}^{4}$ | Medium |
| 2 | $4.05$ ($2.81-5.97$) | $7.79$($5.95-10.2$)$\times{10}^{-6}$ | $1.08$ ($0.88-1.34$) | $3.23$($1.71-4.66$)$\times{10}^{4}$ | Rapid |
| 3 | $3.92$ ($1.89-6.35$) | $8.27$($6.08-10.9$)$\times{10}^{-6}$ | $1.30$ ($1.07-2.06$) | $3.43$($1.89-5.44$)$\times{10}^{4}$ | Rapid |
| 4 | $4.08$ ($2.80-6.05$) | $7.93$ ($6.07-10.3$)$\times{10}^{-6}$ | $1.27$ ($0.99-1.92$) | $3.31$($2.07-6.37$)$\times{10}^{4}$ | Rapid |
| 7 | $3.92$ ($2.45-5.36$) | $7.81$($5.82-11.2$)$\times{10}^{-7}$ | $0.91$ ($0.80-1.29$) | $3.28$($1.65-5.27$)$\times{10}^{4}$ | Medium |
| 8 | $3.77$ ($2.34-5.64$) | $8.04$($5.57-10.9$)$\times{10}^{-6}$ | $0.75$ ($0.67-0.84$) | $3.28$($1.95-5.82$)$\times{10}^{4}$ | Medium |
| 10 | $3.90$ ($2.59-5.85$) | $7.74$($5.60-11.4$)$\times{10}^{-7}$ | $0.49$ ($0.46-0.55$) | $3.18$($1.75-6.22$)$\times{10}^{4}$ | Slow |
| 14 | $4.06$ ($2.97-6.17$) | $7.95$($5.89-11.1$)$\times{10}^{-6}$ | $1.28$ ($1.02-1.75$) | $3.28$($1.95-5.82$)$\times{10}^{4}$ | Rapid |
| Korea | | | | | |
| 13 | $3.93$ ($2.39-6.18$) | $8.02$ ($5.89-10.4$)$\times{10}^{-6}$ | $0.99$ ($0.79-1.36$) | $3.31$($1.58-5.97$)$\times{10}^{4}$ | Rapid |
| 15 | $3.91$ ($2.13-6.10$) | $7.83$($5.80-11.1$)$\times{10}^{-7}$ | $0.78$ ($0.55-0.93$) | $3.24$($1.94-6.09$)$\times{10}^{4}$ | Medium |
| Singapore | | | | |  |
| 2 | $3.78$ ($2.47-6.02$) | $7.96$($6.15-10.6$)$\times{10}^{-6}$ | $0.61$ ($0.51-0.68$) | $3.26$($1.83-5.90$)$\times{10}^{4}$ | Slow |
| 3 | $4.00$ ($2.75-6.36$) | $7.74$($5.77-10.4$)$\times{10}^{-6}$ | $0.33$ ($0.24-0.38$) | $3.26$($1.93-5.62$)$\times{10}^{4}$ | Slow |
| 4 | $3.83$ ($2.24-6.46$) | $7.88$($6.00-10.5$)$\times{10}^{-6}$ | $0.62$ ($0.47-0.69$) | $3.25$($1.82-5.46$)$\times{10}^{4}$ | Slow |
| 6 | $3.86$ ($2.42-6.43$) | $7.93$($5.47-11.0$)$\times{10}^{-6}$ | $0.37$ ($0.26-0.43$) | $3.28$($2.01-6.64$)$\times{10}^{4}$ | Slow |
| 8 | $3.76$ ($2.45-5.87$) | $8.04$($6.01-10.3$)$\times{10}^{-6}$ | $0.41$ ($0.35-0.45$) | $3.34$($1.63-6.28$)$\times{10}^{4}$ | Slow |
| 9 | $3.60$ ($2.39-4.94$) | $8.13$($6.00-10.5$)$\times{10}^{-6}$ | $0.27$ ($0.22-0.31$) | $3.28$($1.61-5.53$)$\times{10}^{4}$ | Slow |
| 11 | $3.94$ ($2.35-6.19$) | $8.04$($6.58-11.2$)$\times{10}^{-6}$ | $1.14$ ($0.90-1.76$) | $3.32$($1.74-6.53$)$\times{10}^{4}$ | Rapid |
| 12 | $3.77$ ($2.05-5.98$) | $8.02$($5.79-11.3$)$\times{10}^{-6}$ | $0.70$ ($0.59-0.84$) | $3.28$($1.85-6.41$)$\times{10}^{4}$ | Medium |
| 14 | $4.03$ ($2.21-7.06$) | $7.52$($5.47-10.6$)$\times{10}^{-6}$ | $0.56$ ($0.45-0.66$) | $3.22$($1.90-6.19$)$\times{10}^{4}$ | Slow |
| 16 | $3.85$ ($2.20-6.35$) | $7.88$($6.04-11.6$)$\times{10}^{-6}$ | $0.46$ ($0.30-0.63$) | $3.25$($1.64-5.59$)$\times{10}^{4}$ | Slow |
| 17 | $3.60$ ($2.19-5.76$) | $8.11$($5.79-10.6$)$\times{10}^{-6}$ | $0.85$ ($0.57-1.25$) | $3.28$($1.71-5.70$)$\times{10}^{4}$ | Medium |
| 18 | $3.63$ ($1.82-6.45$) | $8.07$($6.23-11.3$)$\times{10}^{-6}$ | $0.34$ ($0.26-0.38$) | $3.28$($1.91-6.14$)$\times{10}^{4}$ | Slow |
| China | | | | | |
| C | $3.80$ ($2.21-6.36$) | $7.97$($6.12-10.5$)$\times{10}^{-6}$ | $0.77$ ($0.33-1.18$) | $3.27$($1.73-5.77$)$\times{10}^{4}$ | Medium |
| D | $3.91$ ($2.13-6.44$) | $7.80$($5.76-11.0$)$\times{10}^{-6}$ | $0.53$ ($0.22-1.14$) | $3.19$($1.98-6.06$)$\times{10}^{4}$ | Slow |
| E | $3.79$ ($1.97-5.66$) | $7.98$($6.00-11.4$)$\times{10}^{-6}$ | $0.70$ ($0.35-0.97$) | $3.27$($1.60-5.48$)$\times{10}^{4}$ | Medium |
| H | $4.52$ ($2.42-5.88$) | $8.01$($5.84-10.9$)$\times{10}^{-6}$ | $1.23$ ($0.57-2.10$) | $3.22$($2.01-5.52$)$\times{10}^{4}$ | Rapid |
| I | $4.08$ ($2.40-6.74$) | $7.67$($5.34-10.1$)$\times{10}^{-6}$ | $0.30$ ($0.16-0.43$) | $3.25$($1.38-5.87$)$\times{10}^{4}$ | Slow |
| L | $3.89$ ($2.30-5.82$) | $7.82$($5.78-10.4$)$\times{10}^{-6}$ | $0.54$ ($0.21-0.94$) | $3.24$($1.80-6.09$)$\times{10}^{4}$ | Slow |
| O | $5.62$ ($2.70-7.00$) | $8.70$($6.20-11.4$)$\times{10}^{-6}$ | $2.29$ ($1.36-4.26$) | $3.64$($2.18-7.07$)$\times{10}^{4}$ | Outlier |
| T | $3.94$ ($2.38-5.96$) | $7.94$($5.66-10.5$)$\times{10}^{-6}$ | $1.02$ ($0.68-1.72$) | $3.25$($1.77-6.08$)$\times{10}^{4}$ | Rapid |
