# Supplementary material for: Detection of significant antiviral drug effects on COVID-19 with reasonable sample sizes in randomized controlled trials: A modeling study
Source: PLoS Med. 2021 Jul 6;18(7):e1003660. doi: 10.1371/journal.pmed.1003660 (PMC8259968; doi:10.1371/journal.pmed.1003660)
Supplement: S3 Table — The fixed effect and the random effect for each parameter were estimated by the nonlinear mixed-effect model. Estimated values with standard errors were summarized. (DOCX) [file pmed.1003660.s007.docx]

| Variables or parameters | Description | Unit | $\vartheta$: Fixed effect (SE)* | $\Omega$: SD of random effect (SE)* |
| --- | --- | --- | --- | --- |
| $f(t)$ | Relative fraction of uninfected target cells at time $t$ to those at time 0 | Unitless (fraction) | --- | --- |
| $V(t)$ | Amount of virus at time *t* | RNA copies/ml | --- | --- |
| $\beta$ | Rate constant for virus infection | (RNA copies/ml)^-1^day^-1^ | $7.95\times{10}^{-6}$ ($1.49\times{10}^{-6}$) | $0.16$ ($0.24$) |
| $\gamma$ | Maximum rate constant for viral replication | Day^-1^ | $3.80$ ($1.95$) | $0.27$ ($0.28$) |
| $\delta$ | Death rate of virus-producing cells | Day^-1^ | $0.68$ ($0.09$) | $0.56$ ($0.09$) |
| $V(0)$ | Amount of virus at time 0 (symptom onset) | RNA copies/ml | $3.27\times{10}^{4}$ ($1.04\times{10}^{4}$) | $0.32$ ($0.25$) |

* The parameter for patient $k$, $\vartheta_{i} \left( =\vartheta{\times e}^{\pi_{k}} \right)$ is represented as a product of $\vartheta$ (a fixed effect) and $e^{\pi_{k}}$ (a random effect). $\pi_{k}$ follows the normal distribution with mean 0 and standard deviation $\Omega$. SE: standard error.
