# Supplementary material for: Detection of significant antiviral drug effects on COVID-19 with reasonable sample sizes in randomized controlled trials: A modeling study
Source: PLoS Med. 2021 Jul 6;18(7):e1003660. doi: 10.1371/journal.pmed.1003660 (PMC8259968; doi:10.1371/journal.pmed.1003660)
Supplement: S4 Table — Sample size was computed for different outcomes (duration of virus shedding and cumulative viral load) under assumed inhibition rate (95% or 99%) and inclusion criteria (0.5 days to 4 days). The underlying data for this figure can be found in S4 Data. (DOCX) [file pmed.1003660.s008.docx]

| Outcome | Inhibition rate | All patients  (no inclusion criteria) | Patients treated within “X days” from the onset of symptoms | | | | |
| --- | --- | --- | --- | --- | --- | --- | --- |
|  |  |  | 0.5 days | 1 days | 2 days | 3 days | 4 days |
| Duration of virus shedding | 95% | 13603 | 249 | 584 | 1717 | 4556 | 3200 |
|  | 99% | 11670 | 166 | 458 | 1462 | 3837 | 2840 |
| Cumulative viral load | 95% | 2811 | 12 | 40 | 209 | 583 | 915 |
|  | 99% | 2554 | 11 | 37 | 192 | 533 | 836 |
