# Supplementary material for: Detection of significant antiviral drug effects on COVID-19 with reasonable sample sizes in randomized controlled trials: A modeling study
Source: PLoS Med. 2021 Jul 6;18(7):e1003660. doi: 10.1371/journal.pmed.1003660 (PMC8259968; doi:10.1371/journal.pmed.1003660)
Supplement: Attachment — Submitted filename: PBP_resubmit.docx [file pmed.1003660.s014.docx]

**Response to the reviewers’ comments**

Shoya Iwanami, Keisuke Ejima, Kwang Su Kim, Koji Noshita, Yasuhisa Fujita, Taiga Miyazaki, Shigeru Kohno, Yoshitsugu Miyazaki, Shimpei Morimoto, Shinji Nakaoka, Yoshiki Koizumi, Yusuke Asai, Kazuyuki Aihara, Koichi Watashi, Robin N. Thompson, Kenji Shibuya, Katsuhito Fujiu, Alan S. Perelson,

Shingo Iwami, & Takaji Wakita

September 16, 2020

We would like to thank the reviewers for their thorough reading of the manuscript and their thoughtful comments and suggestions. In this letter, we present the referees’ comments in black and provide our responses in red. All changes made in addressing the reviewers’ comments have been incorporated in the revised version of the manuscript, and we believe that the manuscript has been considerably improved by these changes. All coauthors have agreed to these corrections.

**Editor Remarks to Author:**

Thank you very much for submitting your manuscript "Rethinking antiviral effects for COVID-19 in clinical studies: early initiation is key to successful treatment" (PMEDICINE-D-20-03078R1) for consideration at PLOS Medicine.

Your paper was evaluated by a senior editor and discussed among all the editors here. It was also discussed with an academic editor with relevant expertise, and sent to independent reviewers, including a statistical reviewer. The reviews are appended at the bottom of this email and any accompanying reviewer attachments can be seen via the link below:

In light of these reviews, I am afraid that we will not be able to accept the manuscript for publication in the journal in its current form, but we would like to consider a revised version that addresses the reviewers' and editors' comments. Obviously we cannot make any decision about publication until we have seen the revised manuscript and your response, and we plan to seek re-review by one or more of the reviewers.

**Reply to Editor Remarks:**

Thank you for encouraging us by providing positive comments and giving us an opportunity to revise our manuscript, which resulted from a collaboration among researchers from very different domains including mathematics, medicine, and public health. We believe our study is part of a new research domain that cannot be pursued without such an interdisciplinary team and we are grateful that we still have a chance to report our results in PLOS Medicine, one of the prominent journals in the medical field. Here, we have summarized the changes from the previous version of our manuscript.

**Major changes in revision:**

We added the following results in the Text and Supplementary Information:

1. Title was changed to “**Detection of significant antiviral drug effects on COVID-19 with reasonable sample sizes in randomized controlled trials: a modeling study combined with clinical data**”.
2. Added all the quantitative findings and a sentence describing the main limitation to the **Abstract**.
3. Added **Author Summary**
4. All **Methods** in the Supporting Information were moved to the **main text**.
5. Added the possibility and limitations of extended models in the **Discussion**.
6. Added the unique points of our study for calculating outcomes and the subsequent sample size by using a mathematical model in the **Discussion**.
7. Added **Table S3** for description of variables and parameters in the model.
8. Added calculated sample size with 95% antiviral effect in the **main text** and **Table S4** to show the sensitivity of the calculation.

**Requests from the editors:**

1. Please address all of the reviewers’ comments below – further consideration of your manuscript will depend on satisfactory resolution of the outstanding concerns.

**Reply to Request 1:**

We present the referees’ comments in black and provide our responses to those comments in red.

2. Please revise your title according to PLOS Medicine's style. Your title must be nondeclarative and not a question. It should begin with main concept if possible. "Effect of" should be used only if causality can be inferred, i.e., for an RCT. Please place the study design ("A randomized controlled trial," "A retrospective study," "A modelling study," etc.) in the subtitle (ie, after a colon).

**Reply to Request 2:**

We updated the title accordingly: “Detection of significant antiviral drug effects on COVID-19 with reasonable sample sizes in randomized controlled trials: a modeling study combined with clinical data”.

3. All authors must declare their relevant competing interests per the PLOS policy, which can be seen here:

https://journals.plos.org/plosmedicine/s/competing-interests

For authors with ties to industry, please indicate whether any of the interests has a financial stake in the results of the current study. Specifically, please mention employment by authors to Science Groove Inc.

**Reply to Request 3:**

The authors declare no conflicts of interest associated with this manuscript. An author, Shingo Iwami, is a research advisor of Science Groove Inc, but has not received any personal payments or promises from the company.

4. Please include line numbers in the margin for all lines, preferably not restarting at 1 on each page.

**Reply to Request 4:**

We added line numbers accordingly.

5. abstract:
   a. Please quantify all of the main results (with 95% CIs and p-values), eg. decay rates, sample sizes.

**Reply to Request 5-a:**

We reported all the quantitative findings both in the abstract and main text as much as possible.

b. In the last sentence of the Abstract Methods and Findings section, please describe the main limitation(s) of the study's methodology.

**Reply to Request 5-b:**

We added a sentence describing the main limitation of this study accordingly.

6. At this stage, we ask that you include a short, non-technical Author Summary of your research to make findings accessible to a wide audience that includes both scientists and non-scientists. The Author Summary should immediately follow the Abstract in your revised manuscript. This text is subject to editorial change and should be distinct from the scientific abstract. Please see our author guidelines for more information:

https://journals.plos.org/plosmedicine/s/revising-your-manuscript#loc-author-summary

**Reply to Request 6:**

We added an Author Summary in our revised manuscript.

7. Please remove spaces from within citation callouts, eg: “...and in vivo models [2,3].”

**Reply to Request 7:**

We removed all of the spaces within citation callouts.

8. Please present and organize the Discussion as follows: a short, clear summary of the article's findings; what the study adds to existing research and where and why the results may differ from previous research; strengths and limitations of the study; implications and next steps for research, clinical practice, and/or public policy; one-paragraph conclusion.

**Reply to Request 8:**

We reorganized the discussion section following the instructions.

**Reviewer #1:**

Iwanami and colleagues use a mathematical modelling approach to predict the necessary sample size in SARS-CoV-2 clinical trials. This is an interesting question and different approach from the usual. The authors develop a model of viral growth and decline based on published data, and try to predict how much viral replication might be blocked by treatment at different times (and how hard it would be to detect this).

【Major issues】

1. The effectiveness of a drug early versus late assumes different levels of ongoing replication are happening at the time. Therefore, growth to peak, when peak occurs, how much replication after peak etc, are major factors. However, the data are almost exclusively on what happens after peak. Supp Fig 1 (which needs to be in the main figures, as it is the foundation of the whole analysis) shows that growth rate data is available for only a very few patients (Singapore 16, France 4, (who reach their peaks at 10 and 4 days post-symptoms), maybe a few others). So the statement "the peak of viral load appeared 2-3 days from symptom onset, which was consistent for all patients" is very misleading. For most patients, they were at their highest level when first sampled (and peak was anytime before that). Thus, all the viral growth parameters are highly questionable, as is any assumption that earlier treatment would help (since the peak may have even been before symptoms).

**Reply to major comment 1:**

For most of the cases, observation started 2-3 days after symptom onset, which is around the timing of peak viral load as we demonstrated. Thus, viral load was at the highest when first sampled as noted by the reviewer. However, this does not necessarily mean that we cannot estimate the growth parameters for those without viral load data before the peak. We first tried to fit a fixed effect model for each individual and realized that this is an issue. Next, we thought of fitting the fixed effect model for all the patients’ data, but then we lose the information on individual variability. Thus, we decided to use the mixed effect model and fit the data from all the patients simultaneously, which enables us to estimate the parameters for those without peak viral load data because the individual growth rate (fixed effect + random effect) is partially explained by the fixed effect estimated from all the patients. Indeed, the mixed-effect modelling approach is commonly used in analyzing viral load data and previously parameters were successfully estimated for SARS-CoV-2 even when viral load data before the peak were not available for some cases [1]. However, the solid curves in the figure are based on the point estimate of the parameters (i.e., best fit curves), so giving the viral load peak timing from such curves might not be appropriate and we decided to delete the sentence. We moved Fig.S1 to the main text (it is currently Fig.1) as was suggested.

2. The model depends on 'target cell limitation' being the only factor determining viral decay. As a result of this assumption, it is assumed that there is very little ongoing infection after the peak (and thus blocking viral replication can have little effect). However, this also assumes that infected cells are very long lived and hardly affected by immune responses. If instead the decay phase is a balance of replication and death, then antiviral treatment may have a much greater effect. However, since as in point 1, nothing meaningful can be said around growth, it is hard to see that any conclusions on viral replication (or its inhibition by treatment) are very reliable.

**Reply to major comment 2:**

We agree that immune response is a potentially important factor for the dynamics of viral load. From the point of view of mathematical modeling, antiviral treatments that inhibit viral replication could influence the viral load even if initiated after the peak of viral load; however, almost all clinical trials with late initiation of therapy have shown no statistically significant difference in duration of virus shedding.

There are several different models that can explain the viral load trajectory other than the model we used. However, we chose this model because it better explains the data. Here we fit two other extended models to the data and compared the BICs of the models. First, we used a virus dynamics model considering an eclipse phase of infection that can slow the viral growth rate. The model has been used for SARS-CoV-2 and other viruses [1, 2]:

$$\frac{df_{T}\left( t \right)}{dt}=-\beta f_{T}\left( t \right)V\left( t \right), \left( \text{R}1-1 \right)$$

$$\frac{df_{I}\left( t \right)}{dt}=\beta f_{T}\left( t \right)V\left( t \right)-kf_{I}\left( t \right), \left( \text{R}1-2 \right)$$

$$\frac{dV\left( t \right)}{dt}=\gamma f_{I}\left( t \right)-\delta V\left( t \right). \left( \text{R}1-3 \right)$$

where $f_{T}\left( t \right)=T\left( t \right)/T\left( 0 \right)$ is the relative density of target cells at time $t$ compared with time 0, $f_{I}\left( t \right)=I\left( t \right)/T\left( 0 \right)$ is that of infected cells in eclipse phase, and $V\left( t \right)$ is the concentration of SARS-CoV-2. Note that we assumed quasi-steady-state assumption (QSSA) for the dynamics of viruses to reduce the number of model parameters. The best-fit curves and the 95% prediction intervals of this model along with observed data are shown in **Fig. R1-1** as below:

**Fig. R1-1. Observed and fitted viral load data for individual patients by using the mathematical model with eclipse phase.** The closed dots correspond to the observed viral load that is the same as the data in the manuscript. The curves and shaded regions correspond to the viral load calculated by mathematical model (Eq. R1-1 – R1-3) with estimated individual parameters and the 95% prediction intervals, respectively. The red dots represent the data at or under the detection limit.

Note that the two parameters $f_{I}\left( 0 \right)$ and $k$ were fixed as $0.1$ and $3$ d^-1^ following what was done in a previous study [1] to reduce the number of parameters. We found that the estimates of $\beta$ and $\gamma$ were negatively correlated. This means that larger $\beta$ (more virus infection) is required when $\gamma$ is smaller (less virus replication) and vice versa to realize the amount of virus production expected from the data. This implies that this model cannot distinguish between the rate of virus infection and the rate of virus production, both of which are associated with the initial growth of viral load.

Second, we used another virus dynamics model with an innate immune response, e.g., interferons (IFNs), that can reduce the rate of viral infection. The model also has been used in previous studies [2]:

$$\frac{df\left( t \right)}{dt}=-\frac{1}{1+\eta F\left( t \right)}\beta f\left( t \right)V\left( t \right), \left( \text{R}1-4 \right)$$

$$\frac{dV\left( t \right)}{dt}=\frac{1}{1+\eta F\left( t \right)}\gamma f\left( t \right)V\left( t \right)-\delta V\left( t \right), \left( \text{R}1-5 \right)$$

$$\frac{dF\left( t \right)}{dt}=sV\left( t \right)-\alpha F\left( t \right), \left( \text{R}1-6 \right)$$

where $f\left( t \right)=T\left( t \right)/T\left( 0 \right)$ is the relative density of target cells at time $t$ compared with time 0, $V\left( t \right)$ is the concentration of SARS-CoV-2, and $F\left( t \right)$ is the concentration of IFNs produced from infected cells. We excluded the eclipse phase of infection in the above for simplification. This model also assumed QSSA. The best-fit curves and the 95% prediction intervals of this model along with the viral load data are shown in **Fig. R1-2**.

Note that the parameters $F\left( 0 \right)$, $\eta$, $s$, and $\alpha$ were set as $1$, $0.1$, $0.01$, and $0.7$, respectively. Given the setting of the model parameters, the estimated values of $\beta$ and $\gamma$ were expected to be higher than those in our model because our model does not account for the effect of IFNs. However, the estimated values were close to our model. Further, we have changed the IFN related parameter values and confirmed consistent results. This modelling exercise implies that the innate immune response to inhibit *de novo* infection does not play an important role in realizing the observed viral load decay. Note that, since $\delta$ (death rate of virus-producing cells) in our simple mathematical model implicitly accounts for immune response by cytotoxic T lymphocytes, we omit discussion on the adaptive immune response in changing $\delta$ here.

**Fig. R1-2. Observed and fitted viral load data for individual patients by using the mathematical model with innate immune response.** The closed dots correspond to the observed viral load that is the same as data in the manuscript. The curves and shaded regions correspond to the viral load calculated by mathematical model (Eq. R1-4 – R1-6) with estimated individual parameters and the 95% prediction intervals, respectively. The red dots represent the data at or under the detection limit.

We summarized the Bayesian Information Criteria (BIC) and Corrected Bayesian Information Criteria (BICc) values calculated from the data fitting with our model and the other two models in **Table R1-1** to compare the goodness-of-fit.

**Table R1-1. Comparison of BIC and BICc between the current model and the two extended models.**

| **Models** | **BIC** | **BICc** |
| --- | --- | --- |
| Our model | 962.0 | 974.6 |
| Model with eclipse phase* | 959.2 | 971.6 |
| Model with innate immune response** | 963.1 | 975.7 |

*$f_{I}\left( 0 \right)$ and $k$ were fixed as 0.1 and 3.

**$F\left( 0 \right)$, $\eta$, $s$ and $\alpha$ were fixed as $1$, $0.1$, $0.01$, and $0.7.$

BIC: Bayesian Information Criteria, BICc: Corrected Bayesian Information Criteria.

We found that the values of BIC and BICc for the three models were close, and the model with eclipse phase is slightly preferred. However, for the models with eclipse phase and innate immune response, we needed to fix several parameters to estimate the other parameters. Because the results are consistent between the models and the other two models required fixing a few parameters given limited data (i.e., only viral load data is available), we believe employing the minimal model is appropriate.

However, we understand that more complex models may be able to better capture the dynamics of viral load change if appropriate scientific evidence becomes available. Thus, we mentioned the possibility of extended models and the limitation of our study in the **Discussion** (Page 24-25, Line 470-481).

3. There seems an assumption that clinical trials would mainly depend on duration of viral shedding as a primary outcome. Mortality is a much more definitive and important outcome. This and other clinical outcomes may well be much more useful than viral load. Indeed, since mortality occurs well after presentation (when viral loads are declining in mild infection, but declining more slowly in severe), this suggests that ongoing infection may play a larger role. In any event, studies of clinical outcome may require far fewer patients - so why would one rely on viral load outcomes (which could only ever be useful as a proxy of clinical outcomes)?

**Reply to major comment 3:**

We agree that mortality is an important and ultimate clinical outcome both at the individual and population level. However, that does not undermine the value of outcomes related to viral load (such as the duration of viral shedding), which have a different interpretation from clinical outcomes. One thing that can be captured by viral load but not by clinical outcome is potential transmissibility. From a clinical viewpoint, each drug has its purpose of use. For example, immunosuppressive agents (e.g., dexamethasone) are expected to reduce clinical symptoms and mortality; meanwhile, the efficacy of antiviral drugs should be evaluated primarily using viral load. In addition, the major objective of therapy depends on severity. Lifesaving is the most important for patients with severe illness. For mild cases, physicians attempt to prevent the condition and spread of infection from getting worse by using drugs with few adverse effects. A primary endpoint should be determined on the basis of the objectives and goals of the clinical trials. As most COVID-19 patients have mild to moderate disease, the duration of viral shedding would be more appropriate than mortality as a primary outcome. Indeed, there are plenty of studies using the duration of viral shedding as an outcome (**Table S1**). We added sentences to justify the outcomes related to viral load in the Methods section (Page 14, Line 262-268).

4. The major conclusion - that duration of viral shedding is highly variable and thus there is low statistical power to detect differences - is an important one. You don't need a model to conclude this (just analysing the variability in duration of detection would be useful). However, the conclusion from this would be that the proxy measure (viral load) is less useful than the most important measure (clinical outcome)?

**Reply to major comment 4:**

Please see **Reply to major comment 3**, where we discussed the importance of viral load as an outcome (viral load could be a proxy measure of clinical outcomes, but itself is an important outcome for sure).

The main reason we used a model is that we cannot compute sample sizes without models. As we demonstrated in our study, the outcomes related to viral load are highly and non-linearly related to the timing of treatment initiation. To make the situation more realistic, we used the observed time delay distribution from symptom onset to treatment initiation (which has huge variability) in our simulations. Even if treatment is initiated at the same time (since symptom onset) in patients, we cannot even reliably guess the duration of viral shedding without the model because the viral dynamics itself is non-linear and there is huge heterogeneity between patients. We believe our approach is not perfect, but is much better than simply assuming an effect size without considering the timing of treatment initiation. We believe this is one of the unique points of our study and is summarized in the discussion section as follows (Page 22-23, Line 422-429):

*“In randomized clinical trials, the calculation of sample size has been performed directly by assuming specific distributions for outcomes with a prespecified effect size. However, as we demonstrated, the antiviral effect is determined not only by dose and type of drug, but also by the timing of treatment initiation and the parameters that govern the virus dynamics. Further, the association between treatment initiation and the outcome (length of viral shedding) is nonlinear; thus, our mathematical model-based approach can provide a more reliable sample size than a conventional effect size-based approach.”*

**Reviewer #2:**

Statistical review of the manuscript 'Rethinking antiviral effects for COVID-19 in clinical studies: early initiation is key to successful treatment' by Shoya Iwanami and colleagues, submitted to PLOS Medicine.

Summary

In this manuscript, Iwanami and colleagues extract data from published longitudinal viral load data of COVID-19 (three studies) and fit a simple within-host model to these data. Using these fits (i.e. parameter estimates), the authors use clustering methods to stratify the viral dynamics as fast, intermediate, and slow. Subsequently, the model armed with parameter estimates is used to gauge the impact of antiviral drugs interfering with viral replication. Based on this, the authors make suggestions as to the sample size needed to detect different in a hypothetical clinical trial.

Evaluation

I like the central idea of the manuscript very much, and believe that approaches such as the one in this manuscript, confronting real-world data with mechanistic within-host models, are highly commendable. HOWEVER, I was considerably less impressed with many details of the study (see below), presentation of the results, and sensitivity analyses. In essence, I would consider the current manuscript an (very interesting) working draft that needs substantial work before it would be suitable for publication. Therefore, I would argue that a rejection with a strong encouragement for resubmission of a thoroughly redrafted manuscript would be appropriate.

**Reply to Reviewer evaluation:**

Thank you for your encouraging but stringent comments, which helped us to rethink how we can effectively deliver the idea, approach, and results to readers. We replied to your comments and questions as below:

【Specific comments】

1. p3, Conclusions. Vague. Try to come up with specific suggestions/insights.

**Reply to major comment 1:**

We apologize that our conclusion was not clear. In this paper there are two main messages for clinical studies of antivirals:

1. Randomization is necessary due to the large variability in viral dynamics.
2. Early treatment initiation is key to observe significant effects of antivirals with true efficacy on mitigating viral replication.

Accordingly, we rephrased the conclusions as follows (Page 5):

“*At present, clinical trials have failed to identify effective antiviral drugs against SARS-CoV-2, and there may be at least two reasons for this: 1) randomization is not performed (i.e., observational studies), and 2) randomization and treatment initiation are delayed. For a statistically significant effect of antiviral drugs on COVID-19 to be observed, a study’s design should consider these two factors.*”

2. p4, l27, "we admit this is not an exhaustive list". Mention all clinical studies (my preferences, as this is so central to the current ms), or drop.

**Reply to major comment 2:**

We deleted the sentence.

3. p4, l36, "truly effective". This requires at the very least mention of an effect size. I found this missing at several other places.

**Reply to major comment 3:**

In the model, we have fixed the antiviral effect in reducing the replication rate, so we cannot define an effect size. However, we think the reviewer means the model and model parameters are not palatable to readers. We have added a table to explain the variables and parameters in the model, the estimated (or assumed) initial conditions, and the parameter values (**Table S3**).

4. p5l 9, "the detection limit". First, I found the description of the data overly short. Second, and more importantly, it is not clear how these the data below the detection limit have been included. The text now suggests that in the estimation procedure these left-censored values have been included as actual measurements, rather than as left-censored values (using cumulative density functions as is standard practice). Please repair if necessary, or the very least explain in more detail.

**Reply to major comment 4:**

We are assuming that “left-censored values” in the reviewer’s comment refers to the values under the detection limit (but still positive), which are typically observed at the late phase of infection. We treated these data as censored data in a “gold standard approach” as is used in such studies. Specifically, the likelihood function reflects the likelihood that the data are in the censoring interval (0 to the detection limit) given parameter values with a right-truncated Gaussian distribution [3]. Accordingly, we added a sentence in the Methods section (Page 13, Line 228-231):

*“To account for data points under the detection limit (see the red dots in Fig. 1), the likelihood function reflected the likelihood that the data are in the censoring interval (0 to the detection limit) given parameter values with a right-truncated Gaussian distribution.”*

5. p5, "Mathematical model". Here a very simple within-host model with target cell limitation is used. Several other models and choices (e.g. wrt functional responses) are possible (e.g., one of the authors, ASP, is an expert on this), and these should be explored, and formally evaluated wrt ability to fit to the data.

**Reply to major comment 5:**

Following your comment, we tested other possible models for data fitting.

First, we used a virus dynamics model considering an eclipse phase of infection that can slow the viral growth rate. The model has been used for SARS-CoV-2 and other viruses [1, 2]:

$$\frac{df_{T}\left( t \right)}{dt}=-\beta f_{T}\left( t \right)V\left( t \right), \left( \text{R}2-1 \right)$$

$$\frac{df_{I}\left( t \right)}{dt}=\beta f_{T}\left( t \right)V\left( t \right)-kf_{I}\left( t \right), \left( \text{R}2-2 \right)$$

$$\frac{dV\left( t \right)}{dt}=\gamma f_{I}\left( t \right)-\delta V\left( t \right). \left( \text{R}2-3 \right)$$

where $f_{T}\left( t \right)=T\left( t \right)/T\left( 0 \right)$ is the relative density of target cells at time $t$ compared with time 0, $f_{I}\left( t \right)=I\left( t \right)/T\left( 0 \right)$ is that of infected cells in eclipse phase, and $V\left( t \right)$ is the concentration of SARS-CoV-2. Note that we assumed quasi-steady-state assumption (QSSA) for the dynamics of viruses to reduce the number of model parameters. The best-fit curves and the 95% prediction intervals of this model along with observed data are shown in **Fig. R2-1** as below:

**Fig. R2-1. Observed and fitted viral load data for individual patients by using the mathematical model with eclipse phase.** The closed dots correspond to the observed viral load that is the same as the data in the manuscript. The curves and shaded regions correspond to the viral load calculated by mathematical model (Eq. R2-1 – R2-3) with estimated individual parameters and the 95% prediction intervals, respectively. The red dots represent the data at or under the detection limit.

Note that the two parameters $f_{I}\left( 0 \right)$ and $k$ were fixed as $0.1$ and $3$ d^-1^ following what was done in a previous study [1] to reduce the number of parameters. We found that the estimates of $\beta$ and $\gamma$ were negatively correlated. This means that larger $\beta$ (more virus infection) is required when $\gamma$ is smaller (less virus replication) and vice versa to realize the amount of virus production expected from the data. This implies that this model cannot distinguish between the rate of virus infection and the rate of virus production, both of which are associated with the initial growth of viral load.

Second, we used another virus dynamics model with an innate immune response, e.g., interferons (IFNs), that can reduce the rate of viral infection. The model also has been used in previous studies [2]:

$$\frac{df\left( t \right)}{dt}=-\frac{1}{1+\eta F\left( t \right)}\beta f\left( t \right)V\left( t \right), \left( \text{R}2-4 \right)$$

$$\frac{dV\left( t \right)}{dt}=\frac{1}{1+\eta F\left( t \right)}\gamma f\left( t \right)V\left( t \right)-\delta V\left( t \right), \left( \text{R}2-5 \right)$$

$$\frac{dF\left( t \right)}{dt}=sV\left( t \right)-\alpha F\left( t \right). \left( \text{R}2-6 \right)$$

where $f\left( t \right)=T\left( t \right)/T\left( 0 \right)$ is the relative density of target cells at time $t$ compared with time 0, $V\left( t \right)$ is the concentration of SARS-CoV-2, and $F\left( t \right)$ is the concentration of IFNs produced from infected cells. We excluded the eclipse phase of infection in the above for simplification. This model also assumed QSSA. The best-fit curves and the 95% prediction intervals of this model along with the viral load data are shown in **Fig. R2-2**.

Note that the parameters $F\left( 0 \right)$, $\eta$, $s$, and $\alpha$ were set as $1$, $0.1$, $0.01$, and $0.7$, respectively. Given the setting of the model parameters, the estimated values of $\beta$ and $\gamma$ were expected to be higher than those in our model because our model does not account for the effect of IFNs. However, the estimated values were close to our model. Further, we have changed the IFN related parameter values and confirmed consistent results. This modelling exercise implies that the innate immune response to inhibit *de novo* infection does not play an important role in realizing the observed viral load decay. Note that, since $\delta$ (death rate of virus-producing cells) in our simple mathematical model implicitly accounts for immune response by cytotoxic T lymphocytes, we omit discussion on the adaptive immune response in changing $\delta$ here.

**Fig. R2-2. Observed and fitted viral load data for individual patients by using the mathematical model with innate immune response.** The closed dots correspond to the observed viral load that is the same as data in the manuscript. The curves and shaded regions correspond to the viral load calculated by mathematical model (Eq. R1-4 – R1-6) with estimated individual parameters and the 95% prediction intervals, respectively. The red dots represent the data at or under the detection limit.

We summarized the Bayesian Information Criteria (BIC) and Corrected Bayesian Information Criteria (BICc) values calculated from the data fitting with our model and the other two models in **Table R2-1** to compare the goodness-of-fit.

**Table R2-1. Comparison of BIC and BICc for the current model and two extended models.**

| **Models** | **BIC** | **BICc** |
| --- | --- | --- |
| Our current model | 962.0 | 974.6 |
| Model with eclipse phase* | 959.2 | 971.6 |
| Model with innate immune response** | 963.1 | 975.7 |

*$f_{I}\left( 0 \right)$ and $k$ were fixed as 0.1 and 3.

**$F\left( 0 \right)$, $\eta$, $s$ and $\alpha$ were fixed as $1$, $0.1$, $0.01$ and $0.7.$

BIC: Bayesian Information Criteria, BICc: Corrected Bayesian Information Criteria.

We found that the values of BIC and BICc for the three models were close, and the model with eclipse phase is slightly preferred. However, for the models with eclipse phase and innate immune response, we needed to fix several parameters to estimate the other parameters. Because the results are consistent between the models and the other two models required fixing a few parameters given limited data (i.e., only viral load data is available), we believe employing the minimal model is appropriate.

However, we understand that more complex models may be able to better capture the dynamics of viral load change if appropriate scientific evidence becomes available. Thus, we mentioned the possibility of extended models and the limitation of our study in the **Discussion** (Page 24-25, Line 470-481).

6. p5, l28-31. The details on virus replication blocking drugs are interesting, but not in the right place in the section on the model.

**Reply to major comment 6:**

We moved the explanation of antiviral drugs to the Introduction.

7. p5, l34. Is the only way drugs can act on viral dynamics. I agree that fairly simple models are to be favored over complex ones, but would suggest that at least some mention should be made of other ways drug action can be incorporated.

**Reply to major comment 7:**

We mentioned how we incorporate different mechanism of action into our simple model as follows (Page 11, Line 201-203):

*“The effect of drugs that blocking de novo infection can be modeled by inhibiting both the* $\beta f\left( t \right)V\left( t \right)$ *and* $\gamma f\left( t \right)V\left( t \right)$ *terms and a drug promoting cytotoxicity can be modeled by increasing* $\delta V\left( t \right)$*, as we discussed in* [4].*”*

8. p6. l5. This is much too short and uninformative, to the extent that it is not possible to reconstruct what the authors have done exactly. The information in the SI only partly helps repair this. Please extend the description. In addition, is there an significant effect of study from which the data were taken? Why not employ a fully Bayesian methods, e.g. using JAGS or Stan? This would result in availability of immediate credible intervals, parameter correlations, etc. Now none of the plots have any indication of the levels of uncertainty surrounding estimates and viral kinetics.

**Reply to major comment 8:**

We apologize for not providing a meticulous explanation of the computational process. We moved details of the parameter estimation process from the SI to the main text (Methods section) and extended the explanation. In brief, we employed a non-linear mixed effect model for parameter estimation. We believe the reviewer’s suggested approach is a fixed effect model. The mixed effect model we used in this study enabled us to estimate both fixed (population) effects and random effects, which explain the individual variability in parameters. This approach is becoming more common than the fixed model approach because viral dynamics has large heterogeneity and the data from each patient are not sufficient to estimate parameters in general[1]. For example, estimation of growth rate for those without data before peak would be impossible with a fixed effect model. We also added 95% CIs for the estimated curves in **Fig. 1**. We guessed the reviewer’s comment, “In addition, is there an significant effect of study from which the data were taken?”, is about the data used in our study and the parameter values for antiviral effect. We used data in which patients were not treated with antivirals to estimate the parameters, as on treatment data is not available. The parameter for the antiviral effect ($\varepsilon$) was fixed and chosen to reflect what we believe are values that antiviral drugs may be able to attain. We mainly varied the antiviral effect ($\varepsilon$) from 95% to 99%, because efficacy is evaluated in large trials only for drugs with sufficient efficacy (i.e., drugs with weak efficacy are not necessarily tested). Thus, we believe the value range we studied is reasonable.

9. p6, l10. Is the clustering robust to other clustering methods than Ward (e.g., single linkage)? Please check.

**Reply to major comment 9:**

We appreciate your suggestion on checking the robustness of the result of the clustering. We performed the clustering with other linkage methods (e.g., single-linkage, average-linkage, and complete-linkage) and obtained qualitatively the same results with average-linkage and complete-linkage in which three clusters were recognized and there were statistically significant differences in $\delta$ among clusters (**Fig. R2-3**). By using the single-linkage method, four groups (i.e., two “rapid decay” groups, a medium group, and a slow group) were recognized excluding a patient (China O). In general, single-linkage clustering is known as being simple, fast, and applicable to a large dataset, but distinct clusters at the early stage are often merged and poorly separated (called the “chaining phenomenon”), so we concluded the result in **Fig. 2** is robust.

**Fig. R2-3. Clustering patients with three linkage methods.** (A) Clustering patients using daily viral load with three linkage methods (e.g., single-linkage, average-linkage, and complete-linkage). (B) Estimated parameters of the mathematical model in the three different groups recognized by using average-linkage and complete-linkage.

10. p7, l11, "The reconstructed viral loads ...". Actually not the loads but the parameter estimates are analyzed by cluster analysis.

**Reply to major comment 10:**

We apologize for the unclear explanation, especially our use of the word “reconstructed.” We certainly analyzed the loads as features by clustering methods. We accordingly updated the description of the process of the cluster analysis as follows (Page 16, Line 282-284):

*“The viral loads over time, which were reconstructed based on the mathematical model with the estimated parameters, were analyzed with a clustering approach”*

11. p7, l20-22. Is there a correlation between groups and symptom scores. Please check the original literature whether this information can be retrieved.

**Reply to major comment 11:**

We appreciate this comment as we initially had the same question. However, we decided not to include the symptom score in the analyses because symptom scores were not consistently available. Further, because we have focused on viral dynamics, we thought using symptom or symptom score might blur the focus of this study.

12. p9. Mention of effect size (now only in SI, and assumed to be 99%) is crucial. Also, exploration of sample size with far less effective drugs is warranted!

**Reply to major comment 12:**

We 100% agree with this comment. Given that the antiviral effect of SARS-CoV-2 is still not well quantified for all candidate drugs, it is fair to compute the sample size for a less effective (but reasonable) antiviral effect ($\varepsilon$ in the model). Accordingly, we have computed the sample size for different $\varepsilon$ (**Table S4**).

13. p10-11. Discussion is a bit superficial, with too many vague/general statements. Try to be precise, and try to single out what makes this study so exciting.

**Reply to major comment 13:**

The reviewer commented on our vague abstract in **comment 1**. We also updated the Discussion section to make our message clear and precise. Briefly, there are two main messages for clinical studies for antivirals:

1. Randomization is necessary due to the large variability in viral dynamics.
2. Early treatment initiation is the key to observing a significant effect of antivirals with true efficacy for mitigating viral replication.

Accordingly, we updated the Discussion section (Page 22-23, Line 406-429).

**Reviewer #3:**

This is a potentially important paper quantifying whether or not the effectivity of novel treatments to COVID-19 can be detected in small groups of patients. By fitting a standard mathematical model to clinical data, the study establishes a marked heterogeneity the viral dynamics among the patients. This study provides suggestions for improvement that would help clinicians to sooner establish the effectivity of treatments.

**Reply to Reviewer summary:**

Thank you very much for clearly summarizing our study. We are also hoping our study will help to improve clinical study design for antiviral drugs. Please find our reply to your comments below.

1. That being said, the manuscript is written in a rather poor style, and this sometimes reduces the clarity of the text. My major recommendation is therefore to markedly improve the text linguistically.

**Reply to major comment 1:**

In light of comments from the other reviewer also, we realize that our manuscript did not efficiently present our findings and messages. We have therefore worked with a science editor to improve the style of the manuscript. We believe our manuscript is now much improved.

2. Secondly, the figures in the main text have no parameter values in their legends, and they contain a lot of useless redundancy (large human figures, no treatment in Panel A is repeated in B).

**Reply to major comment 2:**

We added more information to the figure legends so that readers can understand the figures without reference to the main text. Individual patient parameters are given in **Table S2** and this is now referred to in the caption to **Fig. 1** that shows the model fit to each patient’s data.

**References**

1.	Gonçalves A, Bertrand J, Ke R, Comets E, de Lamballerie X, Malvy D, et al. Timing of antiviral treatment initiation is critical to reduce SARS-CoV-2 viral load. CPT Pharmacometrics Syst Pharmacol. 2020. Epub 2020/06/20. doi: 10.1002/psp4.12543. PubMed PMID: 32558354; PubMed Central PMCID: PMCPMC7323384.

2.	Baccam P, Beauchemin C, Macken CA, Hayden FG, Perelson AS. Kinetics of influenza A virus infection in humans. J Virol. 2006;80(15):7590-9. Epub 2006/07/15. doi: 10.1128/JVI.01623-05. PubMed PMID: 16840338; PubMed Central PMCID: PMCPMC1563736.

3.	Adeline Samson, Marc Lavielle, France Mentré. Extension of the SAEM algorithm to left-censored data in nonlinear mixed-effects model: Application to HIV dynamics model. Computational Statistics & Data Analysis. 2006;51(3):1562-74. doi: 10.1016/j.csda.2006.05.007.

4.	Kim KS, Ejima K, Ito Y, Iwanami S, Ohashi H, Koizumi Y, et al. Modelling SARS-CoV-2 Dynamics: Implications for Therapy. medRxiv. 2020:2020.03.23.20040493. doi: 10.1101/2020.03.23.20040493.
