# Supplementary material for: Detection of significant antiviral drug effects on COVID-19 with reasonable sample sizes in randomized controlled trials: A modeling study
Source: PLoS Med. 2021 Jul 6;18(7):e1003660. doi: 10.1371/journal.pmed.1003660 (PMC8259968; doi:10.1371/journal.pmed.1003660)
Supplement: Attachment — Submitted filename: response_revision_final.docx [file pmed.1003660.s015.docx]

**Response to the reviewers’ comments**

Shoya Iwanami, Keisuke Ejima, Kwang Su Kim, Koji Noshita, Yasuhisa Fujita, Taiga Miyazaki, Shigeru Kohno, Yoshitsugu Miyazaki, Shimpei Morimoto, Shinji Nakaoka, Yoshiki Koizumi, Yusuke Asai, Kazuyuki Aihara, Koichi Watashi, Robin N. Thompson, Kenji Shibuya, Katsuhito Fujiu, Alan S. Perelson, Shingo Iwami, Takaji Wakita

February 17, 2021

We would like to thank the reviewers for their thorough reading of the manuscript and their thoughtful comments and suggestions. In this letter, we present the referees’ comments in black and provide our responses in red. All changes made in addressing the reviewers’ comments have been incorporated in the revised version of the manuscript. We also updated the dataset used in this study as we found that we had included two patients from France who were taking antivirals. We now have replaced them with two SARS-CoV-2 patients from Korea. We updated the analysis excluding the two French cases and including two Korean patients. Despite this change in two subjects all of our major findings have been retained. The only difference from the previous version was that one patient from China (China O) was an outlier in the cluster analysis as indicated in the revised **Fig. S2B**. **Figures 1-5**, **S1-S4** and **Tables S2-S4** have been updated. All coauthors have agreed to these corrections.

Reviewer #4

**> In this manuscript, the authors focused on the factors which might affect the power of clinical studies for antiviral drugs against SARS-CoV-2. Longitudinal viral load data from three published studies were extracted and fitted in a virus dynamics model. They report two essential factors in clinical studies: heterogeneity in virus dynamics among patients and late timing of treatment initiation. If patients could be early recruited in randomized controlled trials, a more significant outcome would be observed even with a sample size. Above all, it is a quite interesting approach with important practical significance. Maybe the model is overly simplistic to mimic the viral growth kinetics after treatment, since there are many interactional factors, such as the exposure dose, colonization, genetic background, immunoregulation, and so on. In general, the later the treatment is introduced, the more factors will show their impact on viral replication, leading to a more confounding outcome. However, this study revealed the key association between the timing of recruiting and viral shedding/ cumulative viral load.**

We would like to thank the reviewer for understanding the scope of our study and providing positive comments. As the reviewer pointed out, there are several limitations associated with the simplicity of the model we used. Regardless, we believe that our approach using a simple viral dynamics model helps design clinical trials of antivirals, as is described in the discussion section (**Lines 472-512, Pages 24-26**).

**> Many adverse outcomes were observed in patients with high viral loads at the initiation of treatment. Several estimated parameters, including , , , and (0), were applied, while the role of viral load at (0) should be evaluated or explained.**

In response to the reviewer’s comment, we have added another paragraph in the discussion section (**Lines 450-461, Pages 23-24**):

“Regarding the association between viral load and clinical outcomes such as mortality and clinical scores, it has been observed in a number of studies that high viral loads are associated with severe clinical outcomes [1-3] and increased risk of mortality [4] . The data we used in this study do not contain clinical outcomes, thus we cannot correlate our results with clinical outcomes. However, we did not find a significant difference in the viral load at symptom onset, $V\left( 0 \right)$, which is typically among the highest viral loads observed in a patient, between groups. The groups we identified were characterized by a difference in the death rate of virus-producing cells which was reflected in the virus decay rate. For prognosis purposes, we need to better understand when and which biomarkers including the viral load differentiate between severe and non-severe cases.”

**References**

1.	Liu Y, Yan L-M, Wan L, Xiang T-X, Le A, Liu J-M, et al. Viral dynamics in mild and severe cases of COVID-19. The Lancet Infectious Diseases. 2020;20(6):656-7. doi: 10.1016/s1473-3099(20)30232-2.

2.	Pujadas E, Chaudhry F, Mcbride R, Richter F, Zhao S, Wajnberg A, et al. SARS-CoV-2 viral load predicts COVID-19 mortality. The Lancet Respiratory Medicine. 2020;8(9):e70. doi: 10.1016/s2213-2600(20)30354-4.

3.	Zheng S, Fan J, Yu F, Feng B, Lou B, Zou Q, et al. Viral load dynamics and disease severity in patients infected with SARS-CoV-2 in Zhejiang province, China, January-March 2020: retrospective cohort study. Bmj. 2020;369:m1443. Epub 2020/04/23. doi: 10.1136/bmj.m1443. PubMed PMID: 32317267; PubMed Central PMCID: PMCPMC7190077 at <www.icmje.org/coi_disclosure.pdf> and declare: support from the China National Mega-Projects for Infectious Diseases and the National Natural Science Foundation of China, for the submitted work; no financial relationships with any organisations that might have an interest in the submitted work in the previous three years; no other relationships or activities that could appear to have influenced the submitted work.

4.	Néant N, Lingas G, Le Hingrat Q, Ghosn J, Engelmann I, Lepiller Q, et al. Modeling SARS-CoV-2 viral kinetics and association with mortality in hospitalized patients from the French COVID cohort. Proceedings of the National Academy of Sciences. 2021;118(8):e2017962118. doi: 10.1073/pnas.2017962118.
