# Supplementary material for: Detection of significant antiviral drug effects on COVID-19 with reasonable sample sizes in randomized controlled trials: A modeling study
Source: PLoS Med. 2021 Jul 6;18(7):e1003660. doi: 10.1371/journal.pmed.1003660 (PMC8259968; doi:10.1371/journal.pmed.1003660)
Supplement: Attachment — Submitted filename: PBP_final.docx [file pmed.1003660.s016.docx]

**Response to the editor’s comments**

Shoya Iwanami, Keisuke Ejima, Kwang Su Kim, Koji Noshita, Yasuhisa Fujita, Taiga Miyazaki, Shigeru Kohno, Yoshitsugu Miyazaki, Shimpei Morimoto, Shinji Nakaoka, Yoshiki Koizumi, Yusuke Asai, Kazuyuki Aihara, Koichi Watashi, Robin N. Thompson, Kenji Shibuya, Katsuhito Fujiu, Alan S. Perelson, Shingo Iwami, Takaji Wakita

May 19, 2021

We would like to thank the editor for their thorough reading of the manuscript and their thoughtful comments and suggestions. In this letter, we present the editor’s comments in black and provide our responses in red. All changes made in addressing the editor’s comments have been incorporated in the revised version of the manuscript. All coauthors have agreed to these corrections.

**Requests from Editors:**

**Abstract:**

**Abstract Background: The final sentence should clearly state the study question.**

We updated the last sentence in Background section to clearly state the study question **(Lines 62-63, Page 4)**.

**Line 62 - please amend to ‘my fail to’**

Thank you. We meant “may fail to”. However, we deleted the sentence as we thought it is redundant.

**Abstract Methods and Findings:**

**Please ensure that it is made clear with the abstract that this is a modelling study.**

We added the sentence to state that our study was a modeling study at the beginning of Methods and Findings section **(Lines 65-66, Page 4)**.

**Please ensure that all numbers presented in the abstract are identical to numbers presented in the main manuscript text - there seems to be some differences in the decay rates presented in the abstract and results (line 300)**

The numbers in Abstract Methods and Findings were the means and 95% CI of the estimated decay rate for three groups, whereas those in the main text were the minimum and maximum of them. To make this clearer, we reported the mean values in the main text **(Lines 302-303, Page 17)**.

**In the last sentence of the Abstract Methods and Findings section, please describe the main limitation(s) of the study's methodology.**

We added a sentence to describe the main limitation of this approach in Abstract Methods and Findings section **(Lines 87-88, Page 5)**.

**Abstract Conclusions:**

**Please begin your conclusions with "In this study, we observed ..." or similar, to summarise your main findings, and go on to emphasize what is new.**

We updated the section accordingly **(Lines 92-95, Page 5)**.

**Author Summary:**

**Within the ‘Why was this study done?’ section, please clearly state the study question.**

We updated the section accordingly **(Lines 101-104, Page 6)**.

**Introduction:**

**Line 161 - please temper assertions of primacy by adding ‘to the best of our knowledge’ or similar**

We updated the sentence accordingly **(Line 164, Page 9)**.

**Methods:**

**Line 269 - please define AUC at first use**

We clarified the definition of AUC **(Line 269, Page 14)**.

**Results:**

**Line 300 - Please ensure that all numbers presented in the abstract are present and identical to numbers presented in the main manuscript text. Please also provide 95% CI as you have for the data in the abstract.**

We apologies for this. We made the numbers in the abstract and the main text identical **(Lines 302-304, Page 17)**.

**References:**

**Please ensure that journal name abbreviations match those found in the National Center for Biotechnology Information (NCBI) databases, and are appropriately formatted and capitalised.**

We updated the reference list accordingly.

**Please remove extra information contained within reference 39**

We removed the extra information.

**Supplementary files:**

**Please provide legends for each individual table in the Supporting Information.**

We added the legends for Table S1-S4.

**Please include information on the date on which the summary of clinical trials for antiviral drugs for SARS-CoV-2 was completed.**

We added the information in the legend of Table S1 as well as in the main text **(Lines 403, Page 21).**
